# Supplementary material for: Footprints to singularity: A global population model explains late 20th century slow-down and predicts peak within ten years
Source: PLoS One. 2021 May 20;16(5):e0247214. doi: 10.1371/journal.pone.0247214 (PMC8136733; doi:10.1371/journal.pone.0247214)
Supplement: Attachment — Submitted filename: rebuttal_plos2.pdf [file pone.0247214.s003.pdf]

Dear PLOS Editor,

Reviewers comments reproduced here:

Comments to the Author

1. If the authors have adequately addressed your comments raised in a previous round of review and you feel that this manuscript is now acceptable for publication, you may indicate that here to bypass the “Comments to the Author” section, enter your conflict of interest statement in the “Confidential to Editor” section, and submit your "Accept" recommendation.

Reviewer #2: All comments have been addressed

Author response:

Thank you!

The revised manuscript contains a postscript, located at the end before References. In the postscript I acknowledge the release of limited 2020 census data and not that it agrees with my predictions.

Chris Bystroff
Rensselaer Polytechnic Institute
Tro NY
