# Supplementary material for: Footprints to singularity: A global population model explains late 20th century slow-down and predicts peak within ten years
Source: PLoS One. 2021 May 20;16(5):e0247214. doi: 10.1371/journal.pone.0247214 (PMC8136733; doi:10.1371/journal.pone.0247214)
Supplement: Attachment — Submitted filename: rebuttal_plos.docx [file pone.0247214.s002.docx]

Dear PLOS Editor,

Reviewers comments reproduced in black. Authors responses in red.

======== Comments of Reviewer #1 =================

Reviewer #1: I have given detailed comments on the paper in the attached pdf. But my overall comments are as follows.

The topic of the paper is of considerable interest.

But it is very poorly presented with material all over the place.

The mathematical model is poorly presented

The analysis of the parameter fitting is not at all clear.

The assumptions of the work are not clearly articulated.

The data that the model is based on is not clear.

I did not enjoy reading this paper as a mathematical modeller as the presentation was so disjointed.

The work needs a complete overhaul. If that is done successfully then it could well be suitable for publication. See my comments in the attached pdf.

The paper is very close to being rejected outright but the topic is a timely one. However, I do not want to see it again. I was very disappointed with the way the modelling was presented and the disjointed nature of the work..

Author: I have answered the reviewers comments below, in red. One of these speaks to the "disjointed" comment here and this has been addressed.

---------------Answers to Reviewer #1 comments in the PDF:

line 37: "What does hard mean here?"

"hard" is used to mean a formal model,as opposed to a conceptual or informal model that does not return a specific prediction. The context of this text is a contrast with "informal, subjective and descriptive models ..."

line 40: "It cannot continue to grow exponentially"

Agreed.

line 99: "What does this mean?" (referring to "subdivide humanity")

This refers to the paper cited, in which the human population is broken down into demographics, either by age or by region, creating more variables and thus a more complex model. I argue that models should be simpler not more complex.

line 110: "dark space" is crossed out.

I think this is a request for me to leave out the drama. OK. Done.

line 113: "What follows is not results. It it mainly methodology."

Referring to the heading "Results". The paper describes a model or methodlogy, which is the "result" of this study. I have seen other headings used to present a computational model, "Approach" for instance. But in this paper I choose to use the conventional heading, because I see the methodology as the result.

line 142: "I assume u is a constant"

Yes, u is a constant. All variables in the model are constants in the course of the simulation. Only stocks vary with time. In parameteric optimization, however, all variables are variable, of course.

line 183: "This needs further amplification and a reference."

Some additional depth has been provided here, including a reference to Lotka-Volterra oscillation. (Anisiu, M. C. (2014). Lotka, Volterra and their model. Didáctica mathematica, 32, 9-17.)

line 187: "Parameter" fitting

Word added.

line 187 "Be clear what the parameters are and their specific ranges. "

All parameters are defined with ranges in Table 1.

line 192: "You should have defined this before" referring to hyper-exponential model.

I think the comment means that the sentence was useful and should be moved up to the beginning of the paper.

line 197: There should be a sensitivity analysis. I see it is done later."

I think these are two comments with the second one having been added later. Yes, sensitivity analysis was done in two different ways. First, I show the sensitivity between four variables in an exhaustive four dimensional search. Second, I did a random search of one million different variable combinations randomly drawn from the defined ranges, showing the combinations that produced a good fit. Sensitivity was done in this way rather than the usual random sampling of ranges because the variables were highly correlated.

line 207 "That is a huge difference. Discuss." referring to the statement "between 1 billion and 6 billion by 2100"

The divergence in the predictions is comparable than those of other population models. For example, the 80% confidence range from (Lutz et al 2001) for the 2100 population is from 5.57 to 12.22 billion, a range of 6.65 billion. The 80% confidence region for World4 in 2100 is 2.2 to 6.3 billion, drawn from the 184 simulations in Figure 1, a range of 4.1 billion. The uncertainty is inherent in the model and the data. Changes in parameters may compensate each other in the 1970-2010 timeframe that was used for fitting, but thereafter the changes may manifest large differences. For example, raising ***E_0_*** (total world biocapacity) while raising ***a*** (system fragility) compensate to give similar outputs for 1970-2010, but after that date the higher ***E_0_*** and ***a*** give a lower predicted population at 2100, and the lower **E_0_** and ***a*** give a higher prediction at 2100. I believe this uncertainty reflects the reality that we don't know the true fragility of the system, nor do we know the true total biocapacity.

line 239: "This is not particulary showing confidence." referring to "...0.5 billion to as many as 6 billion. Longer term predictions are less confident"

The stated range is wide (essentially 100%) confidence interval. In the new text I present numbers for the 80% confidence interval, which is narrower.The statement "longer term predictions are less confident" is meant to say the model may be increasingly wrong in the more distant future. It may be that we no longer behave like a K-selected species in the future. If so, then these calculations will be wrong, since the model assumes population will always rise to the carrying capacity. In fact, a non-business-as-usual scenario is presented that explores the possibility that humanity takes more control over its numbers in the future.

line 283: "This seems very unlikely" referring to "peaks at or around 2022".

Since the year 2022 is fast approaching and we have not read about the peaking of population in the news one might assume the prediction must be wrong. I humbly submit that we may not actually know the current population of humans on the Earth. We knew it in 2010 and it was 6.9 billion. Every number you may have heard for the years since 2010 has been a projection using one of the most popular models. As you see in the paper, the projection of (Lutz, 2001) is a long, slowing growth trajectory, based on TFR, LEB and migration. Lutz's model is lacking any attempt to model the food supply, water availability or any such global limit that would push the numbers down. Current population that we hear about in the news is always an extrapolation. Alternate models, especially that of Meadows et al, are not given a voice in the media, for reasons unknown. Indeed, a peak in 2022 seems unlikely if one blindly accepts (Lutz 2001), but I ask that my work not be judged against flawed models that are assumed correct.

lines 299-312: "This is very subjective, for example, arguably a move away from animal based food production can be very significant."

My model is a business-as-usual projection. A world in which we suddenly stop eating meat, while possible, falls into the "wishful thinking" category of modeling that I set out to avoid. Even so, acceding to curiosity, I did add a non-BAU projection which we "control" our own population. The reader could interpret this as a world without animal-based food production because that would indeed free up more of Nature to perform ecosystem services and thereby reduce human impact. With regard to subjectivity, the text in this paragraph is only interpretive of the model results. At no point did current events factor into the design or fitting of the model. The model is objective, not subjective, in the sense that the numbers are derived from numerical methods only. Any subjectivity is confined to the way the results are interpreted. But I still feel that the a real-world illustration of a population approaching its peak is useful to the reader, albeit subjective.

line 326: "Who could have foreseen the very rapid development of the COVID19 vaccines?" referring to "... successful treatment of disease requires..."

Good question. I see it as the culmination of centuries of exponential growth in technology. On close inspection, the new vaccines are not quantum leaps in science but just natural extensions of existing technology. COVID19 is a clear example of an unanticipated event that increased the death rate, revealing that the current technology was obsolescent. At a higher population we live more densely and mix more, giving a virus has a much greater opportunity to spread than it did 50 years ago. Our SARS antiviral technology was absent. But the gap in technology was quickly filled by government action and vaccine development.

line 357: "I still do not get a full view of the mathematical model."

As one who teaches a course in system dynamics, I have learned that systems are best understood empirically, by changing variables and seeing the outcome in the simulation. It is not possible for me to require the reader to "play" with the model, but the model is freely available and may be cloned within InsightMaker so that anyone can modify it if they are sufficiently interested. A true critical test of the model will come only from usage by experts. I hope that the written part of this work is intriguing enough to get modelers interested in playing with the model.

To satisfy the reviewer I have gone over the Methods section again to improve the presentation and make it clearer. Changes are in red. I have rearranged text and added subheadings.

line 369: "The structure of this paper is vary strange. Results before model description does not help in reading this paper."

This is a very important insight for me. I put the Methods after Results because this is common in my field, and because a previous reader asked for it, saying he got lost in the methods development without knowing the outcomes. I thought it would make more sense to present the output of the model before delving into the mechanics of how it was developed. But I have taken this suggestion and have made extensive changes to clarify meaning and guide the reader.

line 376: "It would be clearer if this were written..."

Log form is suggested for Eq 6. Yes, that makes sense.

line 376: "A possibility to get a better fit..."

The suggestion is to use the form c exp(d t^alpha) instead of c exp(d t).

I don't know whether this will improve the fit, but it would go against my principles to add more variables than necessary. The fit is as good as it can get and I see no correlated residual that would justify adding another variable. In a later comment, it is asked whether there are too many variables. That is likely. I am looking into decreasing the number of variables.

line 379: "why that only?" referring to "except 2010"

Perhaps this was confusingly worded. The hyper-exponential equation fits the data increasingly poorly as we go from 1980 to 2010, reinforcing the need for additional parameters to model the downward discrepancy. In the best fit version of Eq 6, discrepancies increase with time and the latest time point, 2010, is off by the most. Obviously Eq 6 is insufficient, and that is the point. A downturn must be added.

line 387: "But you say later model (6) only accurate to 1960. Many inconsistencies."

I have tracked down the inconsistencies and made them consistent. The reason for inconsistency of the inconsistency of the date is that the deviation is gradual not abruspt. Thus it is somewhat arbitrary to pick a date at which the Eq 6 model became wrong.

line 498: "It is not clear how all these values in (7) through (10) interact with (6). The model seems poorly set out." referring to lines 408 to 497.

I have made an effort to make the make the layout of the model more clear.

line 523: "The model is over-specified. There are too many parameters. Hence the problems with parameter fitting."

The non-linear least-squares fitting algorithm "Solver" failed to converge when more than 3 variables were fitted, probably due to high covariance. This happened because of weaknesses in the algorithm, not because the model was over-specified. Solver uses the simplex method or a genetic algorithm, but does not use a gradient based method must less a conjugate gradient, which would have succeeded, perhaps. This problem was easily solved by writing the fortran program Hyperfit, an interactive multidimensional least-squares minimizer. Its exhaustive search method avoids any convergence issues. With regard to overparameterization, I am sensitive to the problem. My approach was to add parameters only where needed and to resist added complexity. Six variables are required for the hyper-exponential growth model (4 starting values for four stocks and 2 intrinsic growth rates), two variables express the feedback strength of the ecosphere on each close subsystem (u and v), and four variables (a, b, c, d) convert ecological footprint values to population through carrying capacity. Only four variables (E0, a, u and v) were fit to recent population data. The greatest uncertainty comes from a variable (I0) that is insensitive to fitting but affects future populations. I0 plays a large role in determining where population will level out, thus we will not know for many years what that value is. The 2020 census data will narrow the range.

line 542: "This description is totally unclear."

I agree. I tried to lump several ideas into one paragraph. Upon reconsideration, I deleted the whole paragraph. It was redundant. I have left the omitted text in the document for now, in red ~~strikeout~~ text.

============ Comments of Reviewer 2 =============

Reviewer #2: This is a fairly standard variation of the 'world model' of the Club of Rome flavor and its may variants over the years.

The notion of 'knowledge and obsolescence' may be slightly novel, but if they are modeled as 'stocks' as they are here, this is no different than a standard economic growth model with investment and depreciation. The mathematical symbols have just been given different names.

AUTHOR comments in red:

I assert that the system I am modeling is not an economic system. At no point am I calculating prices of commodities. I am naive on the subject of economics. It is not my field. I am a biologist and I view this work as ecology. I am studying humans as animals with special properties (i.e. technology).

There is a long-standing controversy with regard to environmental modeling, whether it belongs in the field of economics or in the field of ecology (biology). Some attempts have been made to reconcile the dispute by inventing ecological economics, encompassing the environment within the economy. A ecologist might encompass the economy within the environment instead, and it would be more scientifically correct. I choose to avoid the subject altogether. The economy in my model exists within a broader term "technology". It is described in terms of its ability to prolong life and grow the "Humansphere". That's where economics starts and stops within this model. I have tried below to understand and compare my work to the cited works, but for me reading economics is like reading a foreign language. I have done my best.

The manuscript misses a very large literature on economic growth and the environment that uses coupled differential equation models (i.e. system dynamics) developed in mathematical bioeconomics. This is not surprising given the 'engineering-system dynamics' tradition from whence this model comes. These two literatures have often missed one another, creating useless discussions (i.e. the conflict/arguments between the Club of Rome lead by D. H. and D.L. Meadows and multiple economists who focus on economic growth such as Robert Solow, Geoffrey Heal, and Joseph Stiglitz in the 1970's and 80's) and multiple re-inventions of the wheel.

Reviewer 2 requests that I compare my work in global ecology to referenced works in the field of economics. I have done my best, but it is a little like asking a chemist to do psychology. Nowhere in my work is there any economics, except when I have used economics in the lay sense to illustrate a point. So I am caught in a bind with regard to Reviewer 2. I cannot be expected to read and understand the papers suggested to me, any more than a chemist could read and understand psychology papers. Economics and ecology are really very different fields. I am a biologist, biochemist, and a computational modeler. I understand system dynamics, statistics and math. I do not understand economics. I believe that humans are animals and behave like animals in the aggregate. I believe studies of humans as a population should be in the same field of study as population studies of other animals --that is, in the field of ecology, not economics. Economics when applied to ecosystems treats humans differently than other animals, a form of "human exceptionalism" which is not scientific.

To be publishable, this manuscript must demonstrate what it adds to this literature.

This paper does not so much add to the literature listed here so much as it brings the findings to a new and different audience. Ecologists have not really been exposed to global human modeling. The work of Wackernagel notwithstanding, there remains a gap between the dynamic models known and accepted in the economics communities (and that includes previous "world" models) and the audience that sees the world as a physical, chemical, biological system. Like me, these ecologists do not generally understand economics. They only understand the global pressures on population in terms of natural resources, pollution, and climate change, not in terms of, for example, commodities, prices and demographics. I believe that by connecting population modeling to physical world modeling (World4 stocks are expressed in global hectares of biocapacity) we see some feedback mechanisms that were obvious but still poorly and non-quantitatively understood.

The other novelty of World4 is the strong prediction of a peak population within a tight range of years. The prediction is strong because the parameters are aggregated. Because of fewer parameters the system is not overfit but robustly fit. And the fit will get better with time because the deviation from hyperexponential growth is increasing. Another factor s that as the time of the peak population approaches, it becomes easier to see it coming in the numbers.

The trajectories shown in Figures 1-4 are very generic - you can find countless versions of these pictures in the literature. Fitting them to historical data is not difficult or particularly interesting. Thus, I suggest the authors carefully articulate what their model adds, taking into account the literature they missed, including

1) Brander, James A., and M. Scott Taylor. "The simple economics of Easter Island: A Ricardo-Malthus model of renewable resource use." American economic review (1998): 119-138.

The analysis of Easter Island (Rapa Nui) has some obvious extrapolations to the globe (an island in space) but it is an isolated event with a short timeframe. I would argue this is not a substitute for a global model.

There are many variations on this. It is essentially a 'world model'.

It is in fact a "world model" and I have named it World4 with that aspiration. The model of Meadows from Limits to Growth, the 2004 Update" was called World3. Clearly this model is not an updated version of World3, which is a hybrid economic/ecological model. Mine is just ecological. I emailed Dennis Meadows out of courtesy to ask if there would be any issue with calling my model World4. He said there was none. He is not endorsing my model by saying this, just saying he has no problem with the use of the name.

2) Anderies, John M. "Economic development, demographics, and renewable resources: a dynamical systems approach." Environment and Development Economics (2003): 219-246.

Again a world model. This one rigorously analyses the 'generic' behavior of all such models. You will see all the trajectories there that appear in this manuscript.

I looked at this paper and could not find examples of population trajectories at all analogous to what I am attempting to publish. This figure shows a graph from that paper showing oscillation and a mapping to parameter space. This is loosely related to a property of my model, which will oscillate under the right circumstance (but not with optimized parameters). This paper is so far outside my field that I cannot read understand it without a long period of reeducation.

3) Beltratti, Andrea. "Growth with natural and environmental resources." New directions in the economic theory of the environment 25 (1997): 7.

4) Beltratti, Andrea. Models of economic growth with environmental assets. Vol. 8. Springer Science & Business Media, 1996.

5) Beltratti, Andrea, Geoffrey M. Heal, and Graciela Chichilnisky. "Sustainable growth and the green golden rule." Available at SSRN 1374662 (1995).

6) Chichilnisky, Graciela, Geoffrey Heal, and Andrea Beltratti. "The green golden rule." Economics Letters 49.2 (1995): 175-179.

The Beltratti papers are beyond my comprehension. I see some functional forms that I am familiar with, but the terminology around these equations is unfamiliar to me.

7) Xepapadeas, Anastasios. "Economic growth and the environment." Handbook of environmental economics 3 (2005): 1219-1271.

In this paper, pollution is modeled in the context of economic growth. Population is not modeled, but is used as an input factor only. Most of this paper is again unintelligible to me as I am not an economist.

and follow up on the many other models cited in those papers.

Until the model is correctly placed in the existing literature and its novel contribution clarified, it is just another variation of hundreds of such models.

My response again is that my work is not in the field of economics. It is population ecology, very distinct from economics or demographics. It fits in the context of the modeling of any animal population as a function of its ecosystem. As such, the reviewer, who is apparently an economist, was unfortunately required to do the impossible and review an ecology paper. Sorry about that!

----------------- end of reviewer #2's comments
